# A comparative study of fetal cardiovascular assessment: utilizing Doppler ultrasound gated MRI and echocardiography with detailed analysis using five axial views

**DOI:** 10.3389/fcvm.2024.1408071

**Published:** 2024-09-23

**Authors:** B. Hergert, M. Tavares de Sousa, J. Herrmann, P. Bannas, L. Huber, S. Götz, K. Hecher, G. Adam, M. Dargahpour Barough, B. P. Schoennagel

**Affiliations:** ^1^ Department of Obstetrics and Fetal Medicine, University Medical Center Hamburg-Eppendorf, Hamburg, Germany; ^2^ Department of Diagnostic and Interventional Radiology and Nuclear Medicine, University Medical Center Hamburg-Eppendorf, Hamburg, Germany

**Keywords:** fetal MRI, fetal echocardiography, five axial views, congenital heart disease, DUS gating MRI, fetal heart

## Abstract

**Objectives:**

To investigate the diagnostic performance of fetal cardiovascular magnetic resonance imaging (MRI) using Doppler ultrasound (DUS) gating for the evaluation of the standardized five axial views in comparison with fetal echocardiography.

**Methods:**

In this prospective study 29 pregnant women (median: 34.4 weeks of gestation) underwent fetal cardiovascular MRI using DUS gating at 3 Tesla. The standardized five axial views in prenatal screening (fetal abdomen, four-chamber view, left ventricular outflow tract, right ventricular outflow tract, and three-vessel view) were independently assessed and analysed by both fetal MRI and fetal echocardiography on the same day. Image analysis included qualitative assessment and quantitative measurements of cardiovascular structures. MR image quality was assessed using a 4-point scale (from 1 = low to 4 = excellent). Postnatal echocardiography was performed for validation.

**Results:**

17/28 fetuses (60.7%) had pathological findings [16 congenital heart defect (CHD), one diaphragmatic hernia] in prenatal echocardiography. One fetus was excluded due to severe motion. Overall sensitivity and specificity in detecting fetal cardiac abnormalities was 88% and 100%, respectively, for fetal MRI and 100% and 100% for fetal echocardiography. MR image quality for evaluation of cardiac structures was high with a mean score of 2.8 (±0.8) (score 4: 15.9%, score 3: 53.8%, score 2: 19.3%, score 1: 11%). Quantitative measurements did not differ between fetal cardiovascular MRI and fetal echocardiography (all *p* > 0.05).

**Conclusion:**

Diagnostic performance of fetal cardiovascular MRI using DUS gating was comparable to fetal echocardiography. Fetal cardiovascular MRI using DUS gating might be a valuable diagnostic adjunct for the prenatal evaluation of CHD.

## Introduction

Congenital heart defects (CHD) account for 1% of all live births (1, 2). Prenatal diagnosis of CHD optimizes perinatal management and improves neonatal outcome for several CHD (3). However, critical heart defects still have a mortality of 18% (4) and require prompt surgery or intervention (5), highlighting the importance of prenatal diagnosis.

Fetal echocardiography is the reference standard for prenatal detection of CHD. With the normal situs and the four-chamber view as the first established diagnostic axial planes (6) a detection rate of CHD of 40%–50% was achieved (7). Inclusion of additional axial views such as the left and right ventricular outflow tracts (LVOT, RVOT) plus the three-vessel (3V) view (8) established the current standard of the five-axial views. These five-axial views are recommended by the International Society of Ultrasound in Obstetrics and Gynecology (ISUOG) guidelines for performing fetal echocardiography (9). Including these planes, the detection rate of CHD increased up to 60%–80% (10).

Oligohydramnios, dorsoanterior positions, late gestational age and obesity are restrictive conditions for fetal echocardiography and may lead to limited results (11–13). In these cases, a second cross-sectional imaging modality is desirable.

Fetal magnetic resonance imaging (MRI) is established as a second-tool approach especially in cases of fetal brain malformations (14, 15). However, its application for cardiovascular imaging is more challenging and just developing.

The technical challenge of fetal cardiovascular MRI is fetal cardiac gating, i.e., to synchronize the heart beat with MR image acquisition. One solution is to apply an external Doppler ultrasound (DUS) device for fetal cardiac gating (16, 17). Fetal cardiovascular MRI using DUS gating was successfully applied for morphological and functional imaging of the fetal cardiovascular system (18–23). However, a systematic approach for assessment of the five axial planes has not been investigated.

The aim of this study was to investigate the diagnostic performance of fetal cardiovascular MRI using DUS gating for the evaluation of the standardized five axial views in comparison with fetal echocardiography.

## Methods

### Study population

In this prospective study, 29 pregnant women underwent DUS gated fetal cardiovascular MRI and fetal echocardiography on the same day. The study group consisted of 17 fetuses with suspected CHD, anomalies of the central nervous system (CNS) (*n* = 5), and intestinal malformation (*n* = 2). Five voluntary pregnant women without evidence of fetal CHD were also enrolled. All pregnant women provided written informed consent prior to the imaging procedures to participate in this study. The local ethical committee granted ethical approval for this study.

### Fetal echocardiography

Fetal echocardiography with color and pulse-wave Doppler ultrasound was performed using a convex curved-array transducer with a frequency of 2.0–6.0 MHz (Voluson E10, GE Healthcare, Solingen, Germany). The five axial views included the transverse view of the fetal abdomen, the four-chamber view, the left outflow tract (LVOT), the right outflow tract (RVOT) and the three-vessel (3V) view according to current guidelines (9).

### Fetal cardiovascular MRI

DUS gated fetal cardiovascular MRI was performed at a 3T scanner (Ingenia Elition, Philips Medical Systems, Best, The Netherlands). Depending on individual preference, the pregnant women were placed in a supine or lateral position. A MR-compatible DUS sensor (Smart-Sync, northh Medical GmbH, Hamburg, Germany) was placed on the maternal abdomen for recording of the fetal heart beat and used for fetal cardiac gating (19).

T2-weighted turbo spin echo (TSE) sequences (repetition time TR = 2,436 ms, echo time TE = 80 ms, flip angle FA = 90°, field of view FOV = 350 × 300 mm, slice thickness = 4 mm, matrix size = 292 × 216, spatial resolution = 1.2 × 1.4 mm) of the fetal thorax were performed in 3 orthogonal planes for planning of the subsequent cardiac cine-sequences. DUS gated fetal cardiovascular MRI was performed using a multi-slice cine balanced steady-state free-precession (cine-bSSFP) sequence in transversal orientation to cover the fetal heart and great thoracic vessels (TR = 4.1 ms, TE = 2 ms, FA = 60°, FOV = 246 × 246 mm, slice thickness = 5 mm, slices = 12, spatial resolution = 1.5 × 1.5 mm, matrix size = 164 × 160, cardiac phases = 20, gap = −1 mm). In accordance to fetal echocardiography, imaging planes were angulated for 5°–10° in antero-posterior orientation (para-transversal). To avoid breathing artifacts, MR image acquisition was performed under maternal breath hold.

### Postnatal echocardiography

Postnatal echocardiography was assessed within 1–3 days postpartum and served as the reference standard for diagnosis.

### Qualitative analysis

#### Image quality

Image quality of fetal cardiovascular MRI was assessed for each of the five axial views using a 4-point scale. In addition, a detailed analysis of diagnostic quality was performed for every cardiac structure/morphology of each axial view using the same 4-point scale: 1 = low quality (anatomical structures not delineable with certainty, strong artifacts), 2 = moderate quality (anatomical structures recognizable with artifacts), 3 = high quality (reliable definition of cardiac structures, minor artifacts), and 4 = excellent quality (reliable definition of cardiac structures without artifacts). Image and diagnostic quality was rated by two readers in consensus (MDB and BS, with 2- and 13-years of experience in cardiovascular MRI).

#### Diagnostic quality

Diagnostic quality was analyzed according to the current ISUOG guidelines (9). The 4-chamber view was evaluated by assessing the heart size, the thorax ratio, the cardiac axis, ventricle and atrium sizes, the foramen ovale, the attachment of leaflet valves, the cardiac crux, Moderator band and the ventricular septum. For the LVOT, the continuity between the ventricular septum and aorta, the origin of the aorta and the aortic valve with their morphology, opening, and movement were analyzed. Likewise, the origin and valves were studied for the evaluation of the RVOT. In the 3V view including the pulmonary artery, aorta and superior vena cava, the sequence of vessels, the size and relationship to each other, and the alignment were reviewed.

All structures from the five axial views were analyzed and defined as either “normal” or “pathological” to calculate sensitivities and specificities for each structure and for each of the five axial views. Two operators, who were blinded to each other's findings, evaluated fetal cardiovascular MRI (MDB, 2 years experience in cardiovascular MRI) and fetal echocardiography (BH, 6 years experience in fetal echocardiography).

### Quantitative analysis

Quantitative measurements were also performed independently by the same two operators for fetal echocardiography and fetal cardiovascular MRI. Ventricular length and width were measured in the end-diastolic four-chamber view. The diameter of the LVOT and RVOT were compared by measuring below each semilunar valve. In the 3V view, the diameters of the pulmonary artery, aorta, and superior vena cava were measured following established recommendations (24).

### Statistics

For statistical analysis, McNemar's-test was used for qualitative analysis. Measurements in fetal cardiovascular MRI and fetal echocardiography were compared and analyzed using the Student's *t*-test. *P* < 0.05 was defined as statistically significant. SPSS Statistics 27 software was used for the statistical analysis.

## Results

One fetal MRI examination was excluded from further analysis due to severe fetal motion resulting in artifacts. In 8 of the remaining 28 cases (28.6%), the DUS sensor had to be repositioned on the maternal abdomen during MR examination due to fetal movement and intermittent loss of cardiac gating signal. Mean gestational age was 34.4 weeks (range: 29–38 weeks). Pregnant women had a mean age of 30.9 years (range: 19–39) and BMI of 28.8 kg/m^2^ (range: 21–38).

### Qualitative analysis

**Figure 1:**
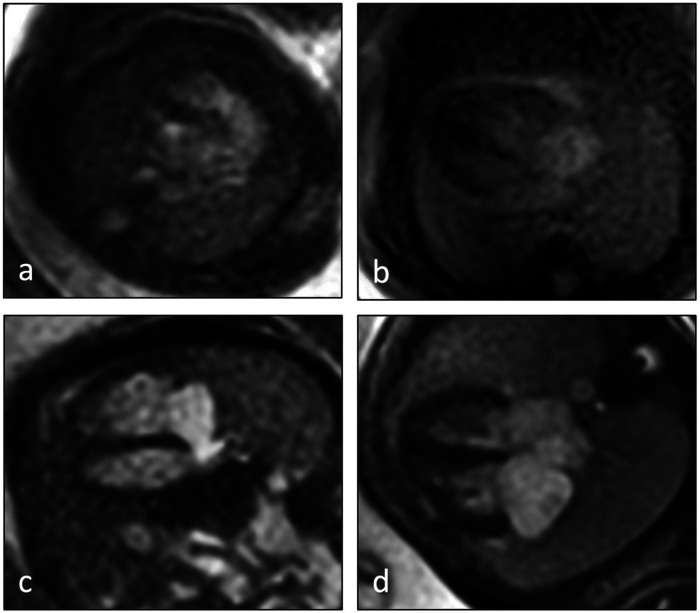
Examples of image quality scores of fetal cardiovascular MRI 4-chamber views according to the applied 4-point scale: **(a)** image score 1 = low quality (anatomical structures not delineable with certainty, strong artifacts), **(b)** image score 2 = moderate quality (anatomical structures recognizable with artifacts), **(c)** image score 3 = high quality (reliable definition of cardiac structures, minor artifacts), **(d)** image score 4 = excellent quality (reliable definition of cardiac structures without artifacts).

Image quality score of fetal cardiovascular MRI, assessed for each of the five axial views per fetus, was high with a mean score of 2.8 ± 0.8 (score 4 in 15.9%, score 3 in 53.8%, score 2 in 19.3%, score 1 in 11%) (Figure 1).

**Figure 2:**
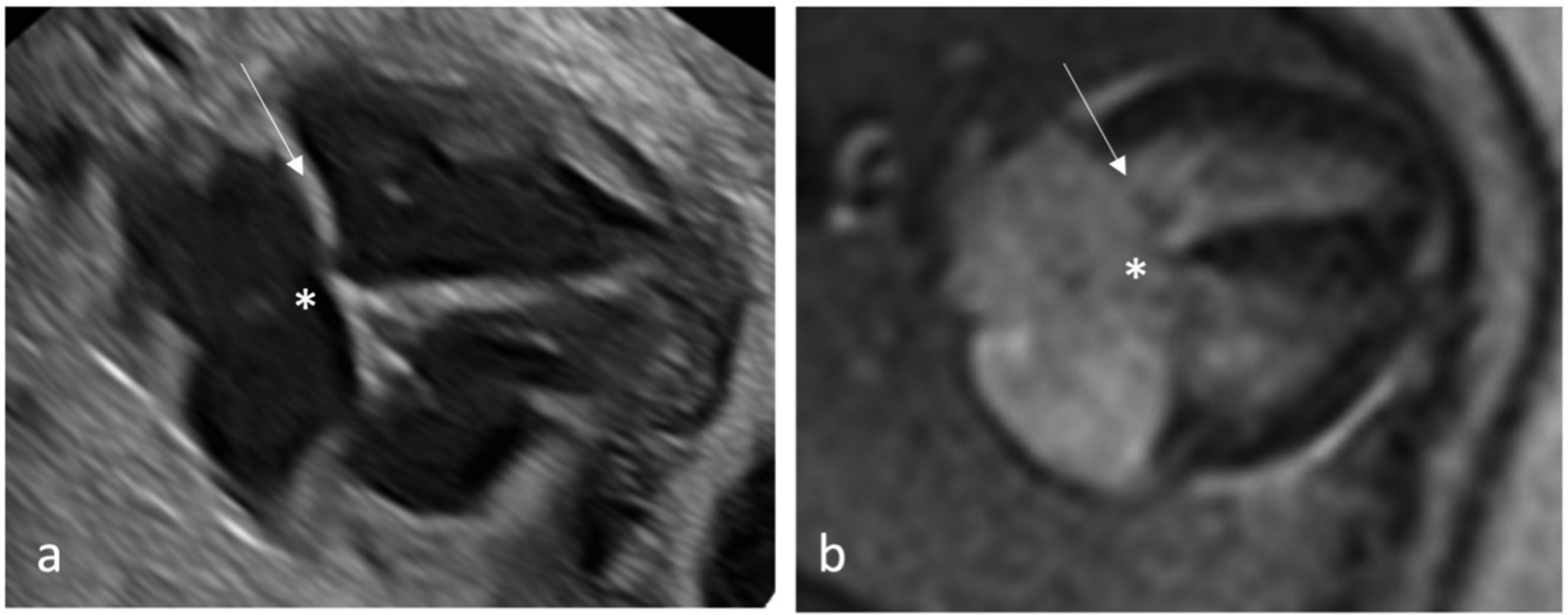
Qualitative assessment of fetal cardiac 4CV on fetal echocardiography **(a)** and DUS gated fetal cardiac MRI **(b)** with intermediate type AVSD detected by both methods. An AVSD, as shown here, is an atrial septal primum defect and an inlet ventricular septal defect (asterisk) with a combination of an abnormal common atrioventricular valve with a linear insertion (arrow).

**Figure 3:**
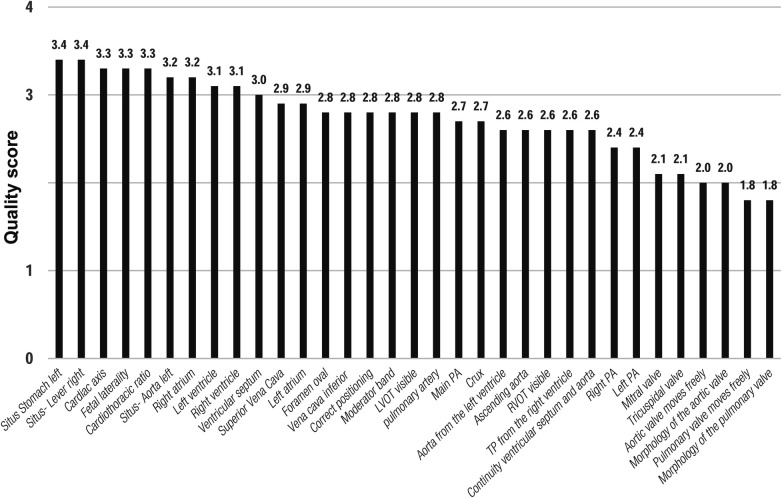
Mean scores for diagnostic quality of fetal cardiovascular MRI for cardiac structures according to the applied 4-point scale. 4 CV, four-chamber view; LVOT, left outflow tract; RVOT, right outflow tract; 3V, three-vessel view; PA, pulmonary artery; VCS, Vena cava superior.

MR diagnostic quality of each anatomical structure/morphology assessed from the five axial views revealed highest scores for cardiac topography and situs and lowest scores for cardiac valves (Figures 2, 3).

A detailed analysis of each cardiovascular structure/morphology for fetal cardiovascular MRI and fetal echocardiography is presented in Table 1. In two cases with prenatal echocardiographic finding of VSD, no VSD was depicted by MRI and postnatal echocardiography. It was not always possible to evaluate all structures in each axial view of the 28 fetuses owing to insufficient image quality (score 1) in MRI or insufficient acoustic window in fetal echocardiography.

**Table 1:** Detailed evaluation of cardiovascular structures and morphology for fetal cardiovascular MRI and fetal echocardiography.

		Echo		MRI	
		Normal (specificity)	Pathologic (sensitivity)	Normal (specificity)	Pathologic (sensitivity)
Situs	Stomach left	27/27 (100%)	1/1 (100%)	27/27 (100%)	1/1 (100%)
	Liver right	28/28 (100%)	–	28/28 (100%)	–
	Aorta left	28/28 (100%)	–	28/28 (100%)	–
	VCI	28/28 (100%)	–	28/28 (100%)	–
	Overall		1/1 (100%)		1/1 (100%)
4CV	Fetal laterality	27/27 (100%)	1/1 (100%)	27/27 (100%)	1/1 (100%)
	Heart occupies a 1/3 of thoracic area	27/27 (100%)	1/1 (100%)	27/27 (100%)	1/1 (100%)
	Cardiac axis	24/24 (100%)	4/4 (100%)	22/24 (92%)	4/4 (100%)
	Left ventricle	21/21 (100%)	7/7 (100%)	21/21 (100%)	4/7 (57%)
	Right ventricle	19/19 (100%)	9/9 (100%)	19/19 (100%)	7/9 (78%)
	Left atrium	26/26 (100%)	2/2 (100%)	26/26 (100%)	1/2 (50%)
	Right atrium	26/26 (100%)	2/2 (100%)	26/26 (100%)	2/2 (100%)
	Moderator band	28/28 (100%)	–	27/27 (100%)	–
	Crux	26/26 (100%)	2/2 (100%)	25/25 (100%)	1/2 (50%)
	Foramen oval	28/28 (100%)	–	26/26 (100%)	–
	Ventricular septum	23/23 (100%)	4/5 (80%)	23/23 (100%)	2/5 (40%)
	Mitral valve	27/27 (100%)	1/1 (100%)	27/27 (100%)	0/1 (0%)
	Tricuspidal valve	27/27 (100%)	1/1 (100%)	27/27 (100%)	1/1 (100%)
	Overall		34/35 (97.1%)		24/35 (68.6%)
LVOT	LVOT visible	26/26 (100%)	2/2 (100%)	22/22 (100%)	2/2 (100%)
	Aorta originates from the left ventricle	27/27 (100%)	1/1 (100%)	25/25 (100%)	1/1 (100%)
	Continuity between the ventricular septum and the anterior wall of the aorta	26/26 (100%)	2/2 (100%)	25/25 (100%)	2/2 (100%)
	Aortic valve moves freely	26/26 (100%)	2/2 (100%)	25/25 (100%)	0/2 (0%)
	Morphology of the aortic valve	26/26 (100%)	1/2 (50%)	26/26 (100%)	0/2 (0%)
	Overall		8/9 (88.9%)		5/9 (55.6%)
RVOT	RVOT visible	28/28 (100%)	–	26/26 (100%)	–
	TP originates from the right ventricle	26/26 (100%)	–	27/27 (100%)	–
	Pulmonary valve moves freely	25/25 (100%)	1/1 (100%)	23/23 (100%)	–
	Morphology of the pulmonary valve	24/24 (100%)	2/2 (100%)	22/22 (100%)	0/1 (0%)
	Main PA	23/23 (100%)	2/3 (67%)	25/25 (100%)	2/3 (67%)
	Right PA	23/23 (100%)	2/2 (100%)	21/21 (100%)	1/2 (50%)
	Left PA	22/22 (100%)	2/2 (100%)	21/21 (100%)	1/2 (50%)
	Overall		9/10 (90%)		4/8 (50%)
3V view	Pulmonary artery	21/21 (100%)	7/7 (100%)	21/21 (100%)	4/6 (67%)
	Ascending aorta	19/19 (100%)	9/9 (100%)	20/20 (100%)	4/6 (67%)
	Superior vena cava	28/28 (100%)	–	26/26 (100%)	–
	Correct positioning	26/26 (100%)	1/2 (50%)	26/26 (100%)	0/1 (0%)
	Relationship	19/19 (100%)	9/9 (100%)	18/18 (100%)	4/8 (50%)
	Overall		26/27 (96%)		12/21 (57%)

4 CV, four-chamber view; LVOT, left outflow tract; RVOT, right outflow tract; 3V, three-vessel view; PA, pulmonary artery; VCS, Vena cava superior. Number of measurements varies from total number of fetuses (*n* = 28) when not all planes could be evaluated for this structure, e.g., due to insufficient image quality (score 1) in MRI or insufficient acoustic window in fetal echocardiography.

The Table 1 above shows an overall sensitivity for the single structures. However, we have also performed an analysis in which the detection of one pathology is sufficient to classify the level as pathological. Then the sensitivity for cardiovascular MRI and fetal echocardiography was 100% and 100% for the situs view, 81% and 94% for the 4-chamber view, 83% and 100% for the LVOT view, 100% and 67% for RVOT view, and 75% and 100% for the 3V view, respectively.

However, overall diagnostic performance, referring to a diagnosis as pathological if at least one axial view revealed a pathological finding, did not differ between fetal cardiovascular MRI und fetal echocardiography. Pathological cases were correctly diagnosed in 15/17 and 17/17 cases by fetal cardiovascular MRI and fetal echocardiography, resulting in a sensitivity of 88% and 100%, respectively. Normal findings were correctly assessed in all fetuses without cardiac abnormalities by both, fetal cardiovascular MRI and fetal echocardiography resulting in a specificity of 100%.

A summary of all cases with prenatal and postnatal findings and neonatal outcome is provided in Table 2.

**Table 2:** Summary of all fetuses with prenatal and postnatal pathologic findings and neonatal outcome**.**

Fetus	GA (weeks)	Situs		4CV		LVOT		RVOT		3V view		Postnatal Echo	Outcome
		Echo	MRI	Echo	MRI	Echo	MRI	Echo	MRI	Echo	MRI		
1	38 + 3	N	N	P	P	N	N	N	N	P	P	Narrow aortic isthmus	Conservative
2	32 + 5	N	N	N	N	P	N	N	N	P	P	Bicuspid aortic valve + aortic stenosis	Intervention
3	33 + 4	N	N	P	P	P	P	N	N	P	P	Hypoplastic aortic arch	Conservative
4	35 + 6	N	N	N	P	P	P	P	P	P	P	Tetralogy of Fallot with high-grade infundibular and valvular pulmonary stenosis. Hypoplastic proximal right pulmonary artery	Surgery
5	33 + 0	N	N	P	P	P	P	N	not evaluable	P	N	Right aortic arch	Conservative
6	35 + 4	N	N	P	P	N	N	N	not evaluable	not evaluable	not evaluable	Ebstein's anomaly Carpentier type C, pulmonary atresia with constricted confluence, ASD of the secondary type	Surgery
7	37 + 1	N	N	P	P	N	N	N	N	N	N	Intermediate type AVSD, or partial AVSD,ASD I,ASD II	Surgery
8	36 + 3	N	N	P	N	N	N	N	N	N	N	Trisomy 13, Microphtalmia Extremely narrow palpebral fissures Left/right shunt over atrial septum, persistent ductus arteriosus with left/right shunt, impaired left ventricular function	No cardial intervention
9	29 + 2	N	N	P	P	N	N	N	N	P	P	CoA,VDS,AVSD, partial Trisomy 8, partial Monosomy 18	Surgery
10	38 + 2	N	N	P	P	N	N	N	N	P	P	Coarctation aortae	Surgery
11	35 + 1	N	N	P	N	P	P	N	N	P	N	Mid-muscular VSD, narrow aortic arch	Conservative
12	34 + 3	N	N	P	P	N	N	N	N	P	N	Interrupted aortic arch type A Multiple collaterals to the descending aorta Atrial septal defect of secondary type	Surgery
13	33 + 0	N	N	P	P	N	N	P	P	P	P	Absent pulmonary valve syndroym, Fallot-Type	Surgery
14	36 + 3	N	N	P	N	N	N	N	N	N	N	Prenatal VSD	Control
15	37 + 6	N	N	P	P	P	P	N	N	P	P	Severe valvular aortic valve stenosis, bicuspid aortic valve Mitral valve dysplasia,left ventricular dilatation with endocardial fibroelastosis	Normal
16	38 + 5	N	N	P	P	N	N	N	N	P	P	Limited hypolplastic aortic arch	Surgery
17	34 + 5	P	P	P	P	N	N	N	N	N	N	Diaphragmatic hernia	Surgery

GA, gestational age; P, pathological; N, normal; 4 CV, four-chamber view; LVOT, left outflow tract; RVOT, right outflow tract; 3V, three-vessel view. ASD, atrial septal defect; AVSD, atrioventricular septal defect; CoA, Coarctation of the aorta; VSD, ventricular septal defect.

In relation to the postnatal cardiac diagnoses, this means for each axial view the following:

The 4CV by fetal cardiovascular MRI revealed 13 anomalies and had one false positive anomalie (case 4) and could not detect the three VSD (case 8,11,15).

The LVOT by fetal cardiovascular MRI revealed 5 anomalies (5/6, 83.3%) and missed an aortic stenosis with a bicuspid aortic valve (case 2).

Prenatal pathological findings of the RVOT in both examination techniques were present. In case 6, pulmonary stenosis was not seen by echocardiography. This plane and a second one was not evaluable on cardiovascular MRI.

The fetal cardiovascular MRI revealed 9 anomalies (9/12, 75%) in the 3V view and could not detected a right aortic arch, an interrupted aortic arch and narrowed aortic arch (case 5,11,12). One fetus could not be evaluated in both modalities.

### Quantitative analysis

**Figure 4:**
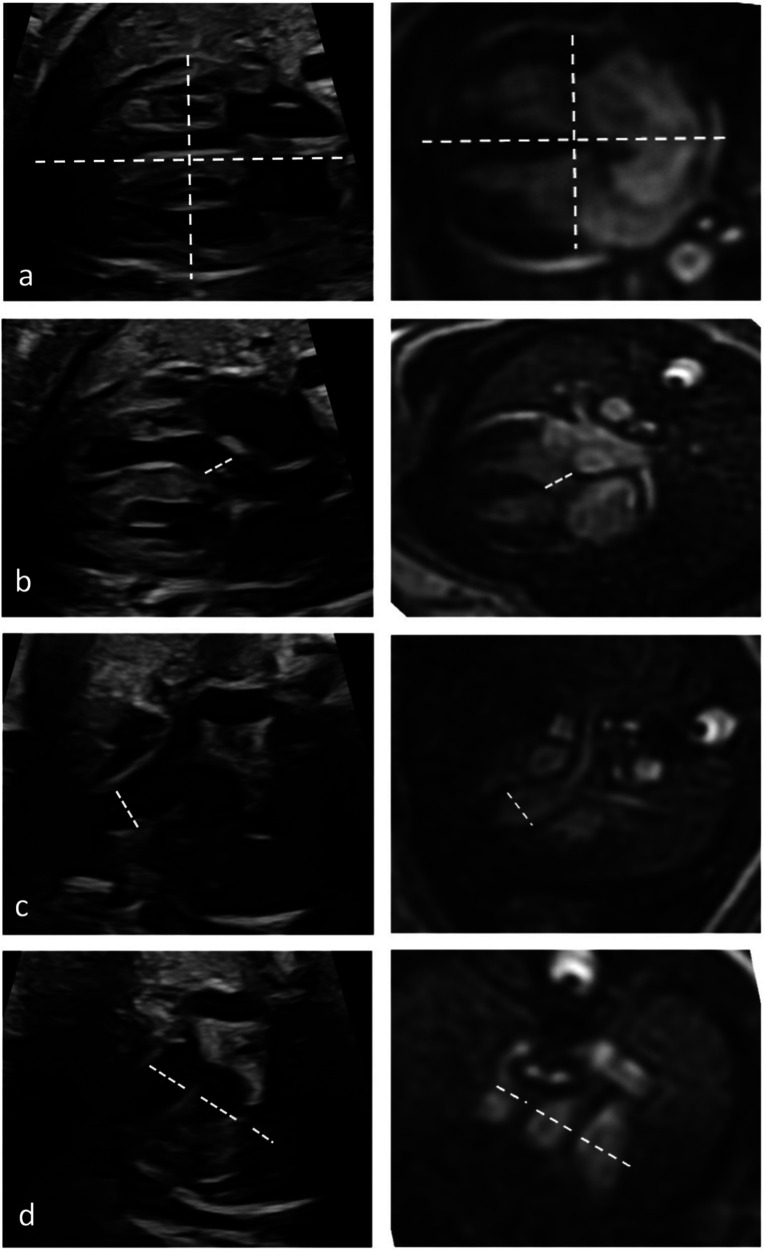
Quantitative assessment of cardiac diameters. Diameter measurements in fetal cardiac five axial views in a healthy Fetus (35 + 1 weeks gestation) in fetal echocardiography (left column) and cardiac MRI (right column). Longitudinal and transverse cardiac diameter from 4-chmaber view **(a)**, diameter of the LVOT **(b)**, the RVOT diameter **(c)** and diameters of pulmonary artery, aorta, and superior vena cava from the three-vessel view **(d)**.

Quantitative evaluation of diameter measurements in the five axial views showed no differences between fetal cardiovascular MRI and fetal echocardiography (Table 3). Examples of measurements are provided in Figure 4.

**Table 3:** Comparison of quantitative measurements for cardiovascular MRI and fetal echocardiography.

	*n*	Echo	MRI	*P*-value
Longitudinal cardiac diameter (mm)	24	44.8 ± 5.6	45.1 ± 5.7	0.71
Transverse cardiac diameter (mm)	25	37.1 ± 5.9	38.4 ± 7.2	0.17
LVOT	23	5.8 ± 0.9	5.4 ± 0.8	0.10
RVOT	22	8.4 ± 1.8	8.6 ± 2.9	0.75
PA	22	8.6 ± 1.9	8.6 ± 1.6	0.70
Aorta	23	5.5 ± 1.4	5.8 ± 1.1	0.08
VCS	21	4.7 ± 0.9	4.7 ± 1.0	0.8

Comparison of measurements were only included in cases where images were available for both methods. LVOT, left outflow tract; RVOT, right outflow tract; PA, pulmonary artery; VCS, Vena cava superior.

## Discussion

Fetal cardiovascular MRI using DUS gating for the assessment of the five axial views revealed similar diagnostic performance in comparison to fetal echocardiography. Our study indicates that the five axial views established for fetal echocardiography can be transferred to fetal cardiovascular MRI for prenatal evaluation of CHD.

Considering the high fetal heart rates and small dimensions of the fetal heart and vessels, cardiac gating is necessary to allow dynamic imaging with high spatial and temporal resolution (19). The method of DUS gating provides a promising approach for anatomical and functional MRI of the fetal cardiovascular system (18–21). An overall high image quality was achieved in this study.

Diagnostic performance of fetal cardiovascular MRI was similar to fetal echocardiography assessing the five axial views. While specificity of MRI was 100% for all five axial views, sensitivities were slightly inferior to fetal echocardiography with 88% vs. 100%, respectively. In two cases a prenatally diagnosed VSD by fetal echocardiography was not depicted by MRI. However, these prenatally described VSDs were not visualized in postnatal echocardiography either. It remains unclear whether MRI revealed false negative, or fetal echocardiography false positive results in these two cases. To note, VSDs may close and disappear during pregnancy or after delivery (25, 26). It must be said that fetal echocardiography has still a better sensitivity at analyzing the single structures, but the MRI achieves high overall diagnostic performance because the detection of one pathology is sufficient to classify the level as pathological. With this increasing improvement in MR imaging, showing in this paper, it can be expected that complete diagnosis of congenital heart defects will be possible in the future.

Evaluation of the cardiac valves revealed to be difficult by cardiovascular MRI, indicated by lower diagnostic quality scores. However, it is also known from adult cardiovascular MRI that fetal echocardiography is superior to MRI in the evaluation of valve abnormalities (27). Difficulties in the detection of specific pathologies may be seen for both, fetal echocardiography and MRI, e.g., Ebstein's anomaly revealed limitations in the interpretability of both imaging modalities due to the enlarged right atrium. Detection of pathology in the 3V view, e.g., evaluation of the relationship of VCI, aorta and main pulmonary artery, with cardiovascular MRI also proved difficult (Table 1). One reason for the lower sensitivity of cardiovascular MRI in the detection of CHD in this study may be the limited experience of the analyzing radiologist with cardiac MRI in general and specifically with CHD. Therefore, detection rates could be higher with increasing routine and expertise in this complex field. However, quantitative analysis demonstrated that fetal cardiovascular MRI is comparable to fetal echocardiography as all diameters were similar for both methods.

This study provides insight into diagnostic performance of MRI in comparison to the reference standard of echocardiography in a fetal population with and without CHD including a detailed analysis of all cardiac and vascular structures. It demonstrated that the evaluation of the five axial views is possible for both imaging techniques including qualitative and quantitative assessment of particular structures. If CHD is suspected by fetal echocardiography after the second trimester scan, MRI may provide a valuable alternative or imaging adjunct because fetal echocardiographic imaging becomes difficult with increasing gestational age and increasing fetal ossification. However, this did not appear to be a limitation in our study.

Fetal cardiovascular MRI has gained substantial interest in recent years, mainly due to technical developments (16). In 2009, Manganaro was one of the first to investigate the performance of fetal cardiac MRI for the detection of CHD, evaluating direct and indirect criteria such as malrotation of the ventricles or cardiomegaly (28). However, assessment of particular anatomical structures was not possible due to the lack of cardiac-gating, which is necessary to allow high spatio-temporal resolutions in fetal cardiovascular MRI (28). Furthermore, no comparison to the reference standard of fetal echocardiography was provided. Dong et al. were able to demonstrate the effective use of fetal cardiac MRI over a period of 5 years and 68 pregnant women with fetuses with a congenital heart defect (29). The detection rate in this study was 79% with MRI and 82% with fetal echocardiography, although no additional cardiac gating or analysis of the 5 axial planes was used (29). They emphasize the examiner's wealth of experience here and also highlight this in the Dong et al. paper from 2020, which could explain the high detection rate even in the early development phase of fetal cardiac MRI (30).

In 2012, Votino et al. examined fetuses with CHD assessing 4-chamber views without cardiac gating (31). The assessed 4-chamber view achieved a comparable sensitivity to our study of 88%. However, for the LVOT and RVOT sensitivities of only 63% and 59% were reported (31). The lacking opportunity to apply fetal cardiac gating at that time could explain the lower sensitivities for LVOT and RVOT in their study. Using DUS-gated cardiovascular MRI we could achieve higher detection rates for the LVOT and RVOT with 83% and 100%, respectively.

Vollbrecht et al. recently investigated 23 fetuses with CHD in a prospective setting and evaluated the diagnostic performance of fetal cardiovascular MRI using DUS-gating, similar to our study (22). Specific structures were analyzed and compared to postnatal findings. High specificity and sensitivity of 99.9% and 91.8% for CHD was found (29). Our results are comparable to Vollbrecht et al., indicating the diagnostic potential of fetal cardiovascular MRI for evaluation and detection of CHD. In addition to that former study we analyzed all cardiac structures referring to current ISUOG guidelines (9). Furthermore, we assessed quantitative measurements of cardiovascular structures, revealing high agreement for fetal echocardiography and MRI. Another noteworthy difference is that in contrast to Vollbrecht et al. the radiologist in our study was blinded to the referral diagnosis.

Nevertheless, a general limitation of fetal MRI is fetal movement causing motion artefacts with resulting low image quality in some of our examined fetuses or in single imaging planes and our study is the relatively small number of included fetuses. The preliminary data suggests that while fetal MRI provides detailed anatomical information, technical challenges such as fetal motion need to be addressed to minimize data loss and ensure reliable cardiac gating. Further studies are also warranted to identify potential subgroups of fetuses that may benefit from additional cardiovascular MRI regarding counseling and neonatal care.

## Conclusion

In conclusion, our study indicates the diagnostic potential of dynamic fetal cardiovascular MRI in the evaluation of the five axial views for prenatal assessment of CHD. In certain conditions, when fetal echocardiography is inconclusive or limited by anatomical or maternal conditions, fetal cardiovascular MRI offers an alternative or adjunct to fetal echocardiography and may therefore improve prenatal assessment of CHD. Our study showed a higher sensitivity for the axial plane of the RVOT in fetal cardiovascular MRI and could be particularly helpful for this question in the diagnosis of CHD. Further studies are warranted to evaluate the clinical impact and influence on fetal outcome of this promising technique.

## Data Availability

The raw data supporting the conclusions of this article will be made available by the authors, without undue reservation.
